# Association of the COVID-19 lockdown with health risk behaviors in South Korean adolescents

**DOI:** 10.1097/MD.0000000000038453

**Published:** 2024-05-31

**Authors:** Chang Hoon Han, Sujin Lee, Jae Ho Chung

**Affiliations:** a Department of Internal Medicine, National Health Insurance Service Ilsan Hospital, Goyang, Republic of Korea; b Department of Neurology, International St. Mary's Hospital, Incheon, Republic of Korea; c Department of Internal Medicine, International St. Mary's Hospital, Catholic Kwandong University College of Medicine, Incheon, Republic of Korea.

**Keywords:** COVID-19, health risk behaviors, Korean adolescents

## Abstract

Since there is no certainty about when the coronavirus disease 2019 (COVID-19) lockdown will be affected by health risk behaviors, so we investigate the effect of COVID-19-related health risk behavior changes using school-based self-reported data from a nationally representative South Korean adolescent population. We analyzed web-based self-reported data from the Korea Youth Risk Behavior Web-based Survey in 111,878 participants (57,069 in COVID-19 prepandemic); 54,809 in during the COVID-19 pandemic. This study included 12 to 18-year-olds. Self-report questionnaires were used to assess socioeconomic status, health risk behaviors, and psychological factors. Health risk behaviors such as alcohol consumption, substance use, and sexual experience significantly decreased in COVID-19 pandemic than in COVID-19 prepandemic. Psychosomatic changes such as stress levels, violence experience, depression, suicidal ideation, suicidal plans, and suicide attempts were significantly lower in COVID-19 pandemic compared to COVID-19 prepandemic (*P* < .001). After adjusting for multiple confounding variables, less alcohol consumption (odds ratio [OR] = 0.98; 95% confidence interval [CI] = 0.88–0.93), less exercise (OR = 0.92; 95% CI = 0.89–0.94), less sexual experience (OR = 0.82; 95% CI = 0.77–0.86), less violence experience (OR = 0.61; 95% CI = 0.55–0.67), less stress (OR = 0.86; 95% CI = 0.84–0.88), less depression (OR = 0.85; 95% CI = 0.83–0.88), less suicidal ideation (OR = 0.93; 95% CI = 0.89–0.97), plans (OR = 0.82; 95% CI = 0.76–0.88), attempts (OR = 0.78; 95% CI = 0.71–0.85) were significantly associated with the COVID-19 pandemic compared to COVID-19 prepandemic. The COVID-19 pandemic was associated with changes in health risk behaviors among Korean adolescents, resulting in alcohol drinking, sexual experience, drug use, violence experience, and suicidal behaviors (idea, plan, and attempts) being decreased during the lockdown period.

## 1. Introduction

In 2020, coronavirus disease 2019 (COVID-19) became a global pandemic, with individual lifestyles being altered ever since. In particular, due to schools being shut during COVID-19, adolescents have had a long period of homeschooling via digital devices.

The COVID-19-induced lockdown might lead to potentially negative effects on health risk behaviors, such as a disruption in daily activities; decreased regular exercise; increased alcohol consumption; and increased anxiety, stress, and depression.^[1]^ During the COVID-19 lockdown, adolescents have also been psychosocially affected due to school closure, decreased contact with school friends, and changes in sleep behaviors.^[2,3]^ Changes in school life, such as online classes and homeschooling, have increased mental problems such as problematic social media use,^[4]^ negative emotions such as anxiety and depression, and worsened psychiatric diseases such as obsessive-compulsive disorder^[5,6]^ and sleep behaviors change.^[7]^ In systemic review, Saulle et al^[8]^ showed the negative impact on students’ mental health associated with school closures and distance learning during pandemic.

Although the COVID-19 lockdown (from January 2020 to May 2023) may affect individuals’ general lifestyle, no studies have investigated the association between COVID-19 lockdown and health risk behaviors in adolescents. Since there is no certainty about when the COVID-19 lockdown will be lifted and how exactly it has affected health risk behaviors, we hypothesized that the COVID-19 lockdown would be associated with negative health risk behaviors. Therefore, our study aimed to determine the effect of COVID-19-related health risk behavior changes using school-based self-reported data from a nationally representative Korean adolescent population.

## 2. Methods

### 2.1. Study participants

We used cross-sectional data from the Korea Youth Risk Behavior Web-based Survey (KYRBWS), comprising an adjusted weighted values sampling method that was described on the KYRBWS website^[9]^ to represent Korean adolescents.

In 2019, the KYRBWS was investigated from June 3 to August 12, which was the period before the COVID-19 pandemic. In 2020, KYRBWS data were collected from August 3 to November 13, which was during the mid-pandemic situation.

The KYRBWS survey aimed to assess Korean adolescents’ health status and health behavior in order to provide basic data for this population. It was composed of a complex survey design with selection probabilities and poststratification. Eight hundred sample schools (400 middle schools, 400 high schools) were selected considering region (17 cities or states), city size (large cities, small cities, and counties), and school type (middle school, general high school, and technical high school). Students who had dyslexia or dysgraphia and students who were absent for an extended period were excluded. Since 2015, ethics approval for the KYRBWS was waived by the Korean Center for Disease Control and Prevention Institutional Review Board. The participants completed self-administered questionnaires online in the school’s computer room, and all of them provided informed consent. Among the 112,251 KYRBWS participants (57,303 in 2019; 54,948 in 2020), those without age information were excluded. Finally, 111,878 participants (57,069 in 2019 [COVID-19 prepandemic]; 54,809 in 2020 [COVID-19 pandemic]) aged 12 to 18 years were included in our study.

### 2.2. Sociodemographic factors

Our study investigated sociodemographic factors (age, sex, family income, living area, living with parents, and academic achievement), psychological factors (self-rated health status, self-rated stress status, and depression), health risk factors (smoking, alcohol consumption, regular exercise, sexual experience, illegal drug experience, suicidal ideation, suicidal plan, and suicide attempts), and comorbidity (asthma, allergic rhinitis, atopy). Academic achievement was self-reported by each student using the question “What do you believe your academic achievement to be in school?.” Student responses were: high academic achievement; middle academic achievement; and low academic achievement. Depression was defined using the Korean version of the World Health Organization Composite International Depression, using the following question, which has been validated for health surveys such as our cross-sectional design^[10]^: “Did you experience more than 2 consecutive weeks where you felt sad, blue, or depressed during the last year?” Participants were identified as having comorbidity (asthma, allergic rhinitis, atopy) if they had been diagnosed by a physician.

### 2.3. Health risk behaviors

Regular exercise was defined as ≥3 times/wk of activity in the past 7 days.^[11]^ Lifetime illegal drug use was assessed with the following question: “Have you ever used drugs that are often used nonmedically (glue, butane gas, stimulants, marijuana, amphetamine, heroin, high-dose cold medicine, or anxiolytics for mood elevation, hallucinations, or excessive diet) for mood elevation, hallucinatory experience, or excessive dieting".^[12]^ Smoking and alcohol use were defined as smoking cigarettes (including e-cigarette) or drinking alcohol for more than 1 day over the last month.^[13]^ Suicidal ideation, suicidal plan, and suicide attempts were assessed if participants had experienced them in the previous year. This indicator was a well-documented predictor of suicide behaviors, being well-validated in a previous study.^[14]^ Violence experience was assessed using the following question: “Do you received hospital treatment for violence victimization; for example, after experiencing physical violence; being threatened; or being ostracized by friends, seniors, or adults?.”^[12]^ Sexual experience was assessed through questions such as whether the subjects had ever engaged in sexual intercourse.^[15]^

### 2.4. Data analysis

The general characteristics between COVID-19 pandemic status and health risk behaviors were compared with Chi-square test with complex sampling Rao–Scott correction, to represent the entire population, as this study was designed to use weighted values. Multiple logistic regression analysis with complex sampling was performed adjusted for sociodemographic variables (age, sex, type of school, family income, living area, living with parents, academic achievement level), sociodemographic variables (smoking, alcohol use, regular exercise, sexual activity, violence experience, illegal drug use), psychological variables (health status, stress, depression, suicidal ideation, and suicide attempts), and comorbidities (asthma, allergic rhinitis and atopic dermatitis). The weights recommended by the KYRBWS were applied, and thus all results are presented as weighted values.^[16]^ Data were analyzed using SPSS Statistics software (version 21.0; IBM Inc., Armonk, NY). *P *< .05 was considered to indicate statistical significance.

## 3. Results

The general characteristics of the study participants are listed in Table 1. The prevalence of allergic rhinitis (35.3% vs 34.8%, *P* = .004) and asthma (7.1% vs 6.2%, *P* < .001) in participants during the COVID-19 pandemic was significantly lower than that of COVID-19 prepandemic, respectively. Contrarily, participants had significantly higher atopy during COVID-19 than COVID-19 prepandemic (22.5% vs 23.3%, *P* < .001).

**Table 1:** General characteristics of participants.

	COVID-19 prepandemic (n = 57,069)	COVID-19 pandemic (n = 54,809)	*P* value
Sex, n (%*)			<.001†
Girl	29,694 (51.9)	28,269 (51.8)	
Boy	27,375 (48.1)	26,540 (48.2)	
Age, mean (SE)	15.0 ± 1.8	15.1 ± 1.8	<.001‡
School, n (%*)			<.001‡
Middle school	29,299 (47.9)	28,928 (49.7)	
Academic high school	22,534 (43.0)	20,932 (41.7)	
Vocational high school	5236 (9.1)	4949 (8.6)	
School type, n (%*)			<.001†
Southern school	9760 (17.2)	9305 (17.3)	
Girl school	9540 (17.0)	9064 (16.2)	
Coeducation	37,769 (65.8)	36,440 (66.5)	
Residence, n (%*)			.05†
Rural	3557 (4.6)	3307 (4.5)	
Urban	53,512 (95.4)	51,502 (95.5)	
Living, n (%*)			<.001†
Living without parents	2960 (4.4)	2574 (3.7)	
Living with parents	54,109 (95.6)	52,235 (96.3)	
Family income, n (%*)			<.001†
Low	7286 (31.8)	7181 (33.0)	
Medium	27,397 (30.2)	26,367 (30.2)	
High	22,386 (38.0)	21,271 (36.8)	
Subjective academic achievement, n (%*)			<.001†
Low	18,061 (31.8)	18,169 (33,0)	
Middle	17,198 (30.2)	16,555 (30.2)	
High	21,810 (38.0)	20,085 (36.8)	
Asthma, n (%*)	3966 (7.1)	3348 (6.2)	<.001†
Allergic rhinitis, n (%*)	19,752 (35.3)	18,527 (34.8)	<.001†
Atopy, n (%*)	12,721 (22.5)	12,597 (23.3)	<.001†

School was categorized as boy only, girls only or coeducation. Academic achievement and family income were assessed by the questions: “How would you rate your academic achievement?,” and “How would you assess the socioeconomic status of your family?.”

COVID-19 = coronavirus disease 2019.

* Estimated mean or rate-adjusted recommended weighted value.

† Chi-square test with Rao–Scott correction, significance at *P* < .05.

‡ Linear regression analysis with complex sampling, significance at *P* < .05.

Table 2 shows the changes in health risk behaviors during the COVID-19 pandemic. Health risk behaviors such as alcohol consumption, substance use, and sexual experience significantly decreased in COVID-19 pandemic than in COVID-19 prepandemic. While smoking did not significantly reduce, regular exercise decreased among participants in the COVID-19 pandemic. Psychosomatic changes after COVID-19 showed positive results. Stress levels, school violence, depression, suicidal ideation, suicidal plans, and suicide attempts were significantly lower in COVID-19 pandemic than in COVID-19 prepandemic (*P* < .001, Table 2).

**Table 2:** Health risk behaviors of COVID-19 status.

	COVID-19 prepandemic (n = 57,069)	COVID-19 pandemic (n = 54,809)	*P* value
Smoking, n (%*)	3370 (6.0)	3152 (5.8)	<.001†
Alcohol, n (%*)	13,809 (24.5)	12,444 (22.8)	<.001†
Regular exercise, n (%*)	19,875 (34.0)	17,657 (31.3)	<.001†
Stress, n (%*)			<.001†
Severe to very severe	22,671 (39.9)	18,595 (34.1)	
Moderate	23,341 (41.0)	24,335 (44.5)	
None to mild	11,057 (19.1)	11,879 (21.4)	
Perceived health status, n (%*)			<.001†
Healthy	40,093 (70.0)	38,355 (69.6)	
Moderate	12,769 (22.6)	12,309 (22.6)	
Bad	4207 (7.4)	4145 (7.8)	
Sexual experience, n (%*)	3168 (5.7)	2446 (4.5)	<.001†
Substance use, n (%*)	540 (1.0)	393 (0.7)	<.001†
Violence experience, n (%*)	1329 (2.3)	692 (1.2)	<.001†
Depression, n (%*)	15,935 (28.1)	13,791 (25.2)	<.001†
Suicidal thoughts, n (%*)	7422 (13.0)	5946 (10.8)	<.001†
Suicidal plans, n (%*)	2255 (3.9)	1933 (3.5)	<.001†
Suicidal attempts, n (%*)	1674 (2.9)	1105 (2.0)	<.001†

Violence experience was assessed using the following question: “Do you received hospital treatment for violence victimization; for example, after experiencing physical violence; being threatened; or being ostracized by friends, seniors, or adults?.”

COVID-19 = coronavirus disease 2019.

* Estimated mean or rate-adjusted recommended weighted value.

† Chi-square test with Rao–Scott correction, significance at *P* < .05.

Table 3 shows the unadjusted and adjusted odds ratio (OR) for health risk behaviors according to the COVID-19 pandemic status in Korean adolescents. Unadjusted OR of alcohol consumption (OR = 0.92; 95% confidence interval [CI] = 0.90–0.95), exercise (OR = 0.89; 95% CI = 0.87–0.92), sexual experience (OR = 0.80; 95% CI = 0.75–0.84), substance use (OR = 0.76; 95% CI = 0.66–0.86), violence experience (OR = 0.54; 95% CI = 0.49–0.59), perceived health status (OR = 0.97; 95% CI = 0.96–0.98), stress (OR = 0.76; 95% CI = 0.74–0.79), depression (OR = 0.86; 95% CI = 0.84–0.88), suicidal ideation (OR = 0.81; 95% CI = 0.79–0.84), suicidal plans (OR = 0.89; 95% CI = 0.84–0.95), and suicide attempts (OR = 0.68; 95% CI = 0.63–0.74) were showed significant chance association with the COVID-19 pandemic period compared to COVID-19 prepandemic. After adjusting for multiple cofounding variables (age, sex, school type, family income, living area, living with parents, academic achievement, self-rated health status, self-rated stress status, depression, and comorbidity [asthma, allergic rhinitis, atopy]), alcohol consumption (OR = 0.98; 95% CI = 0.88–0.93), exercise (OR = 0.92; 95% CI = 0.89–0.94), sexual experience (OR = 0.82; 95% CI = 0.77–0.86), violence experience (OR = 0.61; 95% CI = 0.55–0.67), stress (OR = 0.86; 95% CI = 0.84–0.88), depression (OR = 0.85; 95% CI = 0.83–0.88), suicidal ideation (OR = 0.93; 95% CI = 0.89–0.97), suicidal plans (OR = 0.82; 95% CI = 0.76–0.88), and suicide attempts (OR = 0.78; 95% CI = 0.71–0.85) were showed significant chance association with the COVID-19 pandemic period compared to COVID-19 prepandemic.

**Table 3:** Odds ratio (95% CI) of health risk behaviors in COVID-19 pandemic based on COVID-19 prepandemic.

	Univariate logistic regression	Multiple logistic regression
Alcohol	0.92 (0.90–0.95)	0.98 (0.88–0.93)
Smoking	0.97 (0.93–1.02)	1.01 (0.96–1.06)
Regular exercise	0.89 (0.87–0.92)	0.92 (0.89–0.94)
Sexual experience	0.80 (0.75–0.84)	0.82 (0.77–0.86)
Substance use	0.76 (0.66–0.86)	0.94 (0.82–1.08)
Violence experience	0.54 (0.49–0.59)	0.61 (0.55–0.67)
Perceived health status	0.97 (0.96–0.98)	0.98 (0.95–1.04)
Stress	0.76 (0.74–0.79)	0.81 (0.79–0.84)
Depression	0.86 (0.84–0.88)	0.85 (0.83–0.88)
Suicidal idea	0.81 (0.79–0.84)	0.93 (0.89–0.97)
Suicidal plan	0.89 (0.84–0.95)	0.82 (0.76–0.88)
Suicidal attempts	0.68 (0.63–0.74)	0.78 (0.71–0.85)

Violence experience was assessed using the following question: “Do you received hospital treatment for violence victimization; for example, after experiencing physical violence; being threatened; or being ostracized by friends, seniors, or adults?.” Multiple logistic regression analysis with complex sampling, significance at *P* < .05; adjusted for age, sex, type of school, family income, living area, living with parents, academic achievement level, smoking, alcohol use, regular exercise, sexual activity, violence experience, illegal drug use, health status, stress, depression, suicidal ideation, and suicide attempts, and comorbidities (asthma, allergic rhinitis and atopic dermatitis).

CI = confidence interval, COVID-19 = coronavirus disease 2019.

## 4. Discussion

Our study findings in a large sample of Korean adolescents showed that health risk behaviors improved with the COVID-19 pandemic (i.e., alcohol drinking, sexual experience, drug use, school violence experience, and suicidal behaviors [ideas, plans, and attempts]).

Our study showed alcohol consumption was decreased during the COVID-19 pandemic due to restrictions to social gatherings and decreased social contact with friends, which is in line with findings from a study conducted in the Netherlands.^[17]^ Korean adolescents may be trying to adopt healthier lifestyle habits during the COVID-19 pandemic.^[18]^ The increasing use of new technologies, such as substituting digital media for alcohol drinking, might be a possible reason for the reduction in this habit over the COVID-19 pandemic.^[19]^ Furthermore, a longer COVID-19 pandemic period might show different stress levels^[20]^ with the deprivation of social interactions decreasing alcohol consumption, as observed in our study.^[21]^ However, previous studies have shown mixed results.^[22,23]^ While alcohol consumption during COVID-19 pandemic might be linked with increased drinking behavior,^[22]^ Evans et al^[23]^ showed a reduction in alcohol drinking over the COVID-19 pandemic, similar to our study findings.^[23]^ This discrepancy in findings suggests that the observed alcohol effects of the various studies depend on participants’ demographic characteristics.^[23]^ Thus, further prospective, well-designed studies are needed to elucidate these findings.

Our study showed decline in regular exercise in COVID-19 pandemic, which is consistent with other studies.^[24–26]^ An Italian study showed that 56% of the participants had decreased physical activity during the lockdown period.^[25]^

Our study also showed that suicidal behaviors (ideas, plans, and attempts) decreased during the COVID-19 pandemic compared to COVID-19 prepandemic. These findings are consistent with those from several high-income countries^[27,28]^ but in contrast with the increase in suicide in Japan.^[29,30]^ Although the COVID-19 pandemic period has mostly had a negative impact on adolescents’ mental health,^[31,32]^ some studies have shown different findings.^[33,34]^ Hu and Qian^[31]^ found that the impact of the COVID-19 pandemic on adolescent mental health had different mental health problems in COVID-19 prepandemic. Cohen et al^[32]^ showed a significant increase in anxiety and depressive symptoms during the COVID-19 pandemic, whereas life stress showed no significant change. The negative impact of the COVID-19 pandemic on adolescent mental health is therefore not consistent; many other factors should be considered in order to understand the diverse effects on mental health. Our study showed that suicidal behaviors (ideas, plans, and attempts) significantly decreased during the COVID-19 pandemic, which was consistent with other studies.^[33,34]^ During the early stages of COVID-19 pandemic, China showed a decrease in suicidal behaviors, which might be due to an emphasis placed on community and family support mechanisms.^[33,34]^ In the early COVID-19 pandemic, the suicide rate in Japan decreased by 14% compared to the same time last year, which comprised the biggest drop in 5 years, despite the fear that COVID-19 pandemic would increase stress.^[35]^ School comprises a pressure for some adolescents, and more family time at home may decrease suicidal behaviors. Methodological diversity, such as different questionnaires assessing suicidal behaviors^[36,37]^ may explain the discrepant findings. These mixed findings suggest that adolescents may have different coping mechanisms during the COVID-19 pandemic, resulting in different mental health needs.^[38]^ Taken together, these findings underscore the importance of continued monitoring of the mental health needs of vulnerable adolescents.

Our study showed that smoking^[39]^ and substance use^[40]^ were very low prevalence, but suicidal behaviors^[27,28]^ were very high prevalence comparing those data with other Countries and other studies.

This study had some limitations. First, the survey was based on a self-reported questionnaire. Although samples representative of the Korean youth population were selected in both years, the study was cross-sectional, and a longitudinal follow-up survey could not be conducted for each person. In addition, the long-term effects of the lockdown on health risk behaviors should warrant a longer follow-up study. Second, socioeconomic and cultural aspects, relatively conservative South Korean culture, could also have influenced the impact of the COVID-19 pandemic on health risk behaviors. In addition, our study design could not establish a cause-and-effect relationship between COVID-19 pandemic and health risk behaviors. Finally, the use of data from self-reported questionnaires in analyses might have introduced recall bias and there could be an underestimation of health risk behaviors.

This study used the KYRBWS data gathered from a nationwide population-based weighted sampling; therefore, our study showed a good representation of the entire Korean adolescent population. Almost all participants had the same ethnic background, which minimized other possible confounding factors.

In conclusion, the COVID-19 pandemic was associated with changes in health risk behaviors among Korean adolescents, resulting in alcohol drinking, sexual experience, drug use, school violence experience, and suicidal behaviors (idea, plan, and attempts) being reduced over the COVID-19 pandemic period.

## Author contributions

**Conceptualization:** Chang Hoon Han, Sujin Lee, Jaeho Chung.

**Data curation:** Sujin Lee.
